# *N*-[(3-Phenyl­sulfanyl-1-phenyl­sulfonyl-1*H*-indol-2-yl)meth­yl]propionamide

**DOI:** 10.1107/S1600536809041518

**Published:** 2009-10-31

**Authors:** M. Umadevi, V. Dhayalan, A. K. Mohanakrishnan, G. Chakkaravarthi, V. Manivannan

**Affiliations:** a Department of Chemistry, Pallavan College of Engineering, Kanchipuram 631 502, Tamilnadu, India; b Department of Organic Chemistry, University of Madras, Guindy Campus, Chennai 600 025, India; c Department of Physics, CPCL Polytechnic College, Chennai 600 068, India; d Department of Research and Development, PRIST University, Vallam, Thanjavur 613 403, Tamil Nadu, India

## Abstract

In the title compound, C_24_H_22_N_2_O_3_S_2_, the phenyl rings form dihedral angles of 75.2 (1) and 86.1 (1)° with the indole ring system. The mol­ecular structure is stabilized by intra­molecular C–H⋯O and N—H⋯O hydrogen bonds. The crystal structure exhibit inter­molecular N—H⋯O and C—H⋯O hydrogen bonds, C—H⋯π and π–π [centroid–centroid distance = 3.748 (1) Å] inter­actions.

## Related literature

For the biological activity of indole derivatives, see: Nieto *et al.* (2005 ▶); Olgen & Coban (2003 ▶). For related structures, see: Chakkaravarthi *et al.* (2007 ▶, 2008 ▶).
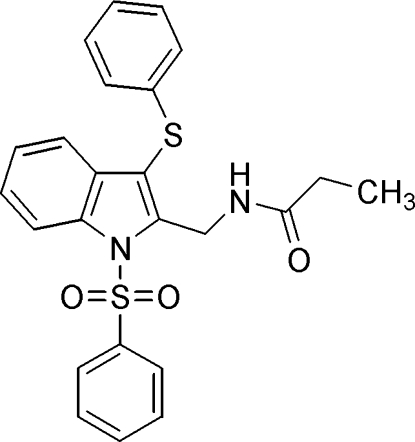


## Experimental

### 

#### Crystal data

C_24_H_22_N_2_O_3_S_2_
*M* *_r_* = 450.56
Monoclinic, 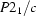

*a* = 10.9216 (3) Å
*b* = 23.1856 (6) Å
*c* = 9.4298 (2) Å
β = 110.147 (1)°
*V* = 2241.74 (10) Å^3^
*Z* = 4
Mo *K*α radiation
μ = 0.27 mm^−1^
*T* = 295 K
0.26 × 0.20 × 0.16 mm

#### Data collection

Bruker Kappa APEXII diffractometer
Absorption correction: multi-scan (*SADABS*; Sheldrick, 1996 ▶) *T*_min_ = 0.934, *T*_max_ = 0.959
26619 measured reflections
5543 independent reflections
3962 reflections with *I* > 2σ(*I*)
*R*_int_ = 0.026

#### Refinement

*R*[*F* ^2^ > 2σ(*F* ^2^)] = 0.041
*wR*(*F* ^2^) = 0.117
*S* = 1.04
5543 reflections
281 parameters
H-atom parameters constrained
Δρ_max_ = 0.22 e Å^−3^
Δρ_min_ = −0.24 e Å^−3^

### 

Data collection: *APEX2* (Bruker, 2004 ▶); cell refinement: *SAINT* (Bruker, 2004 ▶); data reduction: *SAINT* (Bruker, 2004 ▶); program(s) used to solve structure: *SHELXS97* (Sheldrick, 2008 ▶); program(s) used to refine structure: *SHELXL97* (Sheldrick, 2008 ▶); molecular graphics: *PLATON* (Spek, 2009 ▶); software used to prepare material for publication: *SHELXL97*.

## Supplementary Material

Crystal structure: contains datablocks global, I. DOI: 10.1107/S1600536809041518/gw2070sup1.cif

Structure factors: contains datablocks I. DOI: 10.1107/S1600536809041518/gw2070Isup2.hkl

Additional supplementary materials:  crystallographic information; 3D view; checkCIF report

## Figures and Tables

**Table 1:** Hydrogen-bond geometry (Å, °)

*D*—H⋯*A*	*D*—H	H⋯*A*	*D*⋯*A*	*D*—H⋯*A*
N2—H2*A*⋯O2	0.86	2.52	2.939 (2)	111
C13—H13⋯O1	0.93	2.37	2.921 (3)	118
C18—H18⋯O2^i^	0.93	2.52	3.317 (3)	144
N2—H2*A*⋯O3^ii^	0.86	2.15	2.899 (2)	145
C16—H16⋯O3^ii^	0.93	2.60	3.416 (3)	147
C10—H10⋯*Cg*2^iii^	0.93	2.81	3.658 (2)	152
C5—H5⋯*Cg*4^iv^	0.93	2.91	3.788 (4)	158

Symmetry codes: (i) 
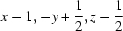
; (ii) 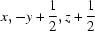; (iii) 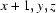; (iv) 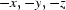. *Cg*2 and *Cg*4 are the centroids of C1–C6 and C15–C20 rings, respectively.

**Fig. 1.**
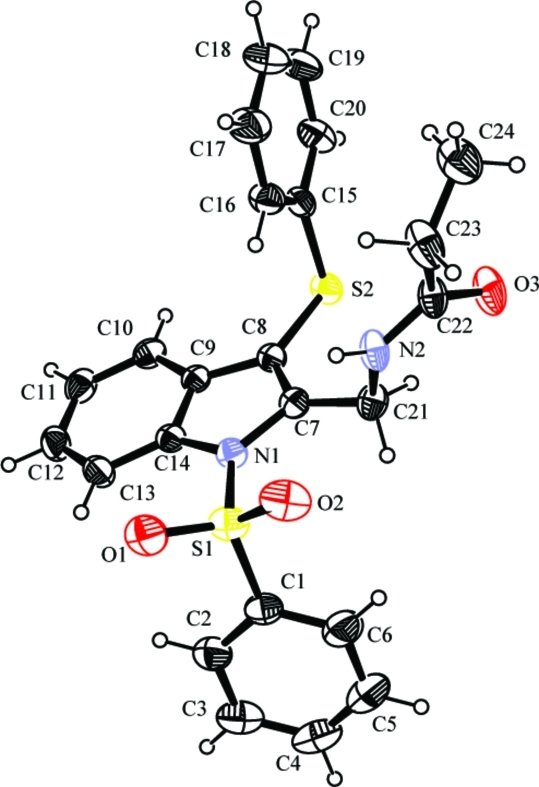
The molecular structure of (I), with atom labels and 30% probability displacement ellipsoids for non-H atoms.

**Fig. 2.**
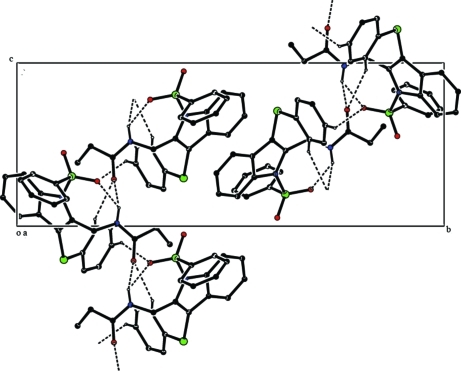
The packing of (I), viewed down the a axis. Hydrogen bonds are shown as dashed lines. H atoms not involved in hydrogen bonding have been omitted.

## supplementary crystallographic information

### Comment

In continuation of our studies of indole derivatives, which are known to exhibit anti-oxidant (Olgen & Coban, 2003) and antibacterial (Nieto *et al.*, 2005) activities, we report the crystal structure of the title compound (I). The geometric parameters in (I) (Fig. 1) agree with the reported values of similar structures (Chakkaravarthi *et al.*, 2007, 2008).

The phenyl rings C1—C6 and C15—C20 form the dihedral angles of 75.2 (1)° and 86.1 (1)°, respectively, with the indole ring system. The mean planes of the two phenyl rings are inclined at an angle of 78.7 (1)° with respect to each other. A distorted tetrahedral geometry [N1- S1- O1 106.3 (1)° and N1—S1—O2 106.4 (1)°] is observed around S1 atom.

The molecular structure is stabilized by intramolecular C—H···O and N—H···O hydrogen bonds and the crystal structure exhibit intermolecular N—H···O and C—H···O hydrogen bonds, C—H···π (Table 1 and Fig. 2) and π···π [*Cg*1···*Cg*3 3.748 (1) Å; symmetry code: -*x*, -*y*, -*z*; *Cg*1 and *Cg*3 are the centroids of N1/C7/C8/C9/C14 and C9—C14 rings, respectively] interactions.

### Experimental

To a solution of 1-phenylsulfonyl-(3-(phenylthio)-2-bromomethylindole (0.5 g, 1.09 mmol) in dry 1,2-dimethoxyethane (20 ml), ZnBr_2_ (0.5 g, 2.22 mmol) and propiononitrile (0.24 g, 4.35 mmol) were added. The reaction mixture was then refluxed for 5 hr under N_2_ atmosphere. It was then poured over ice-water (30 ml) containing 1 ml of conc.HCl, extracted with CHCl_3_ (3 *X* 10 ml) and dried (Na_2_SO_4_). Removal of solvent followed by recrystallization from CDCl_3_ afforded the compound.

### Refinement

H atoms were positioned geometrically and refined using riding model with C—H = 0.93Å and *U*_iso_(H) = 1.2Ueq(C) for aromatic C—H, N—H = 0.86Å and *U*_iso_(H) = 1.2Ueq(N) for N—H, C—H = 0.97Å and *U*_iso_(H) = 1.2Ueq(C) for CH2, C—H = 0.96Å and *U*_iso_(H) = 1.5Ueq(C) for CH3.

### Crystal data

**Table:**

C_24_H_22_N_2_O_3_S_2_	*F*(000) = 944
*M**_r_* = 450.56	*D*_x_ = 1.335 Mg m^−^^3^
Monoclinic, *P*2_1_/*c*	Mo *K*α radiation, λ = 0.71073 Å
Hall symbol: -P 2ybc	Cell parameters from 9917 reflections
*a* = 10.9216 (3) Å	θ = 2.5–27.5°
*b* = 23.1856 (6) Å	µ = 0.27 mm^−^^1^
*c* = 9.4298 (2) Å	*T* = 295 K
β = 110.147 (1)°	Block, colourless
*V* = 2241.74 (10) Å^3^	0.26 × 0.20 × 0.16 mm
*Z* = 4	

### Data collection

**Table:**

Bruker Kappa APEXII diffractometer	5543 independent reflections
Radiation source: fine-focus sealed tube	3962 reflections with *I* > 2σ(*I*)
graphite	*R*_int_ = 0.026
ω and φ scans	θ_max_ = 28.3°, θ_min_ = 1.8°
Absorption correction: multi-scan (*SADABS*; Sheldrick, 1996)	*h* = −14→14
*T*_min_ = 0.934, *T*_max_ = 0.959	*k* = −30→30
26619 measured reflections	*l* = −12→12

### Refinement

**Table:**

Refinement on *F*^2^	Primary atom site location: structure-invariant direct methods
Least-squares matrix: full	Secondary atom site location: difference Fourier map
*R*[*F*^2^ > 2σ(*F*^2^)] = 0.041	Hydrogen site location: inferred from neighbouring sites
*wR*(*F*^2^) = 0.117	H-atom parameters constrained
*S* = 1.04	*w* = 1/[σ^2^(*F*_o_^2^) + (0.0466*P*)^2^ + 0.7649*P*] where *P* = (*F*_o_^2^ + 2*F*_c_^2^)/3
5543 reflections	(Δ/σ)_max_ < 0.001
281 parameters	Δρ_max_ = 0.22 e Å^−^^3^
0 restraints	Δρ_min_ = −0.24 e Å^−^^3^

### Fractional atomic coordinates and isotropic or equivalent isotropic displacement parameters (Å^2^)

**Table:**

	*x*	*y*	*z*	*U*_iso_*/*U*_eq_	
C1	0.35657 (17)	0.09160 (9)	0.2609 (2)	0.0566 (5)	
C2	0.3938 (2)	0.03731 (10)	0.3194 (3)	0.0700 (6)	
H2	0.3548	0.0203	0.3824	0.084*	
C3	0.4893 (2)	0.00865 (12)	0.2834 (3)	0.0863 (8)	
H3	0.5157	−0.0279	0.3226	0.104*	
C4	0.5455 (2)	0.03392 (15)	0.1901 (4)	0.0951 (9)	
H4	0.6101	0.0144	0.1660	0.114*	
C5	0.5080 (3)	0.08729 (16)	0.1321 (4)	0.1013 (9)	
H5	0.5466	0.1039	0.0683	0.122*	
C6	0.4135 (2)	0.11678 (12)	0.1672 (3)	0.0823 (7)	
H6	0.3882	0.1534	0.1280	0.099*	
C7	0.02638 (16)	0.12894 (7)	0.03684 (18)	0.0424 (4)	
C8	−0.08307 (16)	0.09744 (7)	−0.02800 (17)	0.0408 (4)	
C9	−0.08564 (16)	0.05099 (7)	0.07163 (18)	0.0400 (4)	
C10	−0.17279 (18)	0.00610 (8)	0.0598 (2)	0.0505 (4)	
H10	−0.2475	0.0026	−0.0252	0.061*	
C11	−0.1468 (2)	−0.03292 (9)	0.1756 (3)	0.0603 (5)	
H11	−0.2040	−0.0633	0.1690	0.072*	
C12	−0.0360 (2)	−0.02736 (9)	0.3024 (2)	0.0621 (5)	
H12	−0.0205	−0.0543	0.3798	0.075*	
C13	0.0514 (2)	0.01642 (9)	0.3174 (2)	0.0562 (5)	
H13	0.1255	0.0197	0.4033	0.067*	
C14	0.02547 (16)	0.05580 (7)	0.19997 (18)	0.0421 (4)	
C15	−0.31688 (17)	0.15356 (8)	−0.1635 (2)	0.0486 (4)	
C16	−0.2896 (2)	0.18462 (9)	−0.0313 (2)	0.0581 (5)	
H16	−0.2073	0.1826	0.0426	0.070*	
C17	−0.3848 (3)	0.21854 (11)	−0.0095 (3)	0.0791 (7)	
H17	−0.3663	0.2396	0.0794	0.095*	
C18	−0.5068 (3)	0.22168 (13)	−0.1175 (4)	0.0935 (9)	
H18	−0.5709	0.2444	−0.1013	0.112*	
C19	−0.5334 (2)	0.19155 (13)	−0.2483 (4)	0.0920 (8)	
H19	−0.6156	0.1942	−0.3222	0.110*	
C20	−0.4396 (2)	0.15706 (10)	−0.2721 (3)	0.0703 (6)	
H20	−0.4589	0.1361	−0.3613	0.084*	
C21	0.0691 (2)	0.18028 (8)	−0.0289 (2)	0.0523 (4)	
H21A	0.1634	0.1802	0.0017	0.063*	
H21B	0.0340	0.1777	−0.1382	0.063*	
C22	−0.0289 (2)	0.27564 (8)	−0.0787 (2)	0.0547 (5)	
C23	−0.0686 (3)	0.32715 (9)	−0.0106 (2)	0.0770 (7)	
H23A	−0.0868	0.3150	0.0786	0.092*	
H23B	0.0043	0.3538	0.0219	0.092*	
C24	−0.1833 (3)	0.35801 (12)	−0.1114 (3)	0.0908 (8)	
H24A	−0.1677	0.3693	−0.2016	0.136*	
H24B	−0.1990	0.3917	−0.0610	0.136*	
H24C	−0.2581	0.3331	−0.1374	0.136*	
N1	0.09434 (14)	0.10500 (6)	0.17978 (16)	0.0455 (3)	
N2	0.02720 (17)	0.23387 (6)	0.01795 (16)	0.0538 (4)	
H2A	0.0391	0.2389	0.1121	0.065*	
O1	0.23581 (16)	0.10952 (8)	0.44854 (16)	0.0793 (5)	
O2	0.24265 (16)	0.18788 (7)	0.2755 (2)	0.0806 (5)	
O3	−0.04305 (18)	0.27293 (7)	−0.21278 (15)	0.0766 (5)	
S1	0.23448 (5)	0.12841 (2)	0.30516 (6)	0.05906 (16)	
S2	−0.20032 (5)	0.10989 (2)	−0.20547 (5)	0.05258 (14)	

### Atomic displacement parameters (Å^2^)

**Table:**

	*U*^11^	*U*^22^	*U*^33^	*U*^12^	*U*^13^	*U*^23^
C1	0.0413 (9)	0.0629 (12)	0.0571 (11)	−0.0080 (8)	0.0062 (8)	−0.0097 (9)
C2	0.0551 (12)	0.0777 (15)	0.0703 (14)	0.0019 (11)	0.0128 (10)	0.0042 (11)
C3	0.0606 (14)	0.0838 (18)	0.105 (2)	0.0139 (13)	0.0163 (14)	−0.0022 (15)
C4	0.0519 (13)	0.119 (2)	0.117 (2)	0.0048 (15)	0.0318 (15)	−0.0191 (19)
C5	0.0679 (16)	0.122 (3)	0.128 (3)	−0.0041 (16)	0.0524 (17)	0.011 (2)
C6	0.0575 (13)	0.0840 (17)	0.108 (2)	−0.0058 (12)	0.0320 (14)	0.0118 (14)
C7	0.0488 (9)	0.0427 (9)	0.0380 (8)	0.0025 (7)	0.0179 (7)	−0.0070 (7)
C8	0.0447 (9)	0.0437 (9)	0.0352 (8)	0.0051 (7)	0.0150 (7)	−0.0045 (7)
C9	0.0434 (8)	0.0399 (8)	0.0405 (8)	0.0062 (7)	0.0194 (7)	−0.0052 (6)
C10	0.0478 (9)	0.0498 (10)	0.0569 (10)	−0.0007 (8)	0.0219 (8)	−0.0082 (8)
C11	0.0690 (13)	0.0462 (11)	0.0784 (14)	0.0009 (9)	0.0418 (11)	0.0024 (10)
C12	0.0798 (14)	0.0528 (11)	0.0636 (12)	0.0179 (10)	0.0373 (11)	0.0167 (9)
C13	0.0605 (11)	0.0618 (12)	0.0454 (10)	0.0146 (9)	0.0169 (8)	0.0075 (8)
C14	0.0443 (9)	0.0442 (9)	0.0400 (8)	0.0073 (7)	0.0172 (7)	−0.0042 (7)
C15	0.0503 (9)	0.0453 (10)	0.0469 (9)	0.0041 (8)	0.0128 (8)	0.0105 (8)
C16	0.0581 (11)	0.0572 (12)	0.0567 (11)	0.0122 (9)	0.0169 (9)	0.0004 (9)
C17	0.0856 (16)	0.0702 (15)	0.0843 (16)	0.0264 (13)	0.0331 (14)	0.0003 (12)
C18	0.0784 (17)	0.0898 (19)	0.114 (2)	0.0439 (15)	0.0348 (17)	0.0238 (17)
C19	0.0615 (14)	0.099 (2)	0.099 (2)	0.0263 (14)	0.0062 (14)	0.0286 (17)
C20	0.0635 (13)	0.0708 (14)	0.0622 (13)	0.0088 (11)	0.0034 (10)	0.0099 (11)
C21	0.0637 (11)	0.0505 (10)	0.0496 (10)	−0.0055 (9)	0.0284 (9)	−0.0068 (8)
C22	0.0824 (14)	0.0484 (10)	0.0374 (9)	−0.0075 (9)	0.0258 (9)	−0.0033 (8)
C23	0.130 (2)	0.0541 (12)	0.0471 (11)	0.0132 (13)	0.0308 (13)	−0.0022 (9)
C24	0.119 (2)	0.0855 (18)	0.0672 (15)	0.0240 (16)	0.0306 (15)	0.0015 (13)
N1	0.0438 (7)	0.0509 (8)	0.0388 (7)	−0.0017 (6)	0.0102 (6)	−0.0052 (6)
N2	0.0867 (12)	0.0439 (8)	0.0348 (7)	−0.0055 (8)	0.0259 (8)	−0.0049 (6)
O1	0.0732 (10)	0.1127 (13)	0.0419 (8)	0.0004 (9)	0.0070 (7)	−0.0195 (8)
O2	0.0718 (10)	0.0555 (9)	0.0969 (12)	−0.0100 (7)	0.0064 (9)	−0.0252 (8)
O3	0.1285 (14)	0.0722 (10)	0.0369 (7)	0.0096 (9)	0.0383 (8)	0.0020 (6)
S1	0.0518 (3)	0.0646 (3)	0.0511 (3)	−0.0045 (2)	0.0054 (2)	−0.0177 (2)
S2	0.0570 (3)	0.0629 (3)	0.0337 (2)	0.0058 (2)	0.01022 (18)	−0.00357 (19)

### Geometric parameters (Å, °)

**Table:**

C1—C6	1.374 (3)	C15—C16	1.380 (3)
C1—C2	1.378 (3)	C15—C20	1.381 (3)
C1—S1	1.750 (2)	C15—S2	1.7748 (19)
C2—C3	1.374 (3)	C16—C17	1.374 (3)
C2—H2	0.9300	C16—H16	0.9300
C3—C4	1.366 (4)	C17—C18	1.372 (4)
C3—H3	0.9300	C17—H17	0.9300
C4—C5	1.358 (4)	C18—C19	1.358 (4)
C4—H4	0.9300	C18—H18	0.9300
C5—C6	1.370 (4)	C19—C20	1.378 (4)
C5—H5	0.9300	C19—H19	0.9300
C6—H6	0.9300	C20—H20	0.9300
C7—C8	1.354 (2)	C21—N2	1.445 (2)
C7—N1	1.410 (2)	C21—H21A	0.9700
C7—C21	1.489 (3)	C21—H21B	0.9700
C8—C9	1.436 (2)	C22—O3	1.222 (2)
C8—S2	1.7457 (16)	C22—N2	1.326 (2)
C9—C10	1.389 (2)	C22—C23	1.489 (3)
C9—C14	1.392 (2)	C23—C24	1.472 (3)
C10—C11	1.370 (3)	C23—H23A	0.9700
C10—H10	0.9300	C23—H23B	0.9700
C11—C12	1.383 (3)	C24—H24A	0.9600
C11—H11	0.9300	C24—H24B	0.9600
C12—C13	1.367 (3)	C24—H24C	0.9600
C12—H12	0.9300	N1—S1	1.6699 (14)
C13—C14	1.388 (2)	N2—H2A	0.8600
C13—H13	0.9300	O1—S1	1.4165 (17)
C14—N1	1.415 (2)	O2—S1	1.4159 (17)
			
C6—C1—C2	120.6 (2)	C15—C16—H16	120.2
C6—C1—S1	119.78 (18)	C18—C17—C16	120.7 (2)
C2—C1—S1	119.61 (18)	C18—C17—H17	119.7
C3—C2—C1	119.2 (2)	C16—C17—H17	119.7
C3—C2—H2	120.4	C19—C18—C17	119.8 (2)
C1—C2—H2	120.4	C19—C18—H18	120.1
C4—C3—C2	120.0 (3)	C17—C18—H18	120.1
C4—C3—H3	120.0	C18—C19—C20	120.5 (2)
C2—C3—H3	120.0	C18—C19—H19	119.7
C5—C4—C3	120.6 (3)	C20—C19—H19	119.7
C5—C4—H4	119.7	C19—C20—C15	120.0 (2)
C3—C4—H4	119.7	C19—C20—H20	120.0
C4—C5—C6	120.4 (3)	C15—C20—H20	120.0
C4—C5—H5	119.8	N2—C21—C7	112.51 (15)
C6—C5—H5	119.8	N2—C21—H21A	109.1
C5—C6—C1	119.2 (3)	C7—C21—H21A	109.1
C5—C6—H6	120.4	N2—C21—H21B	109.1
C1—C6—H6	120.4	C7—C21—H21B	109.1
C8—C7—N1	108.15 (15)	H21A—C21—H21B	107.8
C8—C7—C21	126.70 (16)	O3—C22—N2	122.52 (18)
N1—C7—C21	125.15 (15)	O3—C22—C23	122.38 (18)
C7—C8—C9	108.81 (14)	N2—C22—C23	115.06 (16)
C7—C8—S2	125.86 (14)	C24—C23—C22	114.89 (19)
C9—C8—S2	125.31 (13)	C24—C23—H23A	108.5
C10—C9—C14	119.86 (16)	C22—C23—H23A	108.5
C10—C9—C8	132.61 (16)	C24—C23—H23B	108.5
C14—C9—C8	107.52 (15)	C22—C23—H23B	108.5
C11—C10—C9	118.87 (18)	H23A—C23—H23B	107.5
C11—C10—H10	120.6	C23—C24—H24A	109.5
C9—C10—H10	120.6	C23—C24—H24B	109.5
C10—C11—C12	120.43 (19)	H24A—C24—H24B	109.5
C10—C11—H11	119.8	C23—C24—H24C	109.5
C12—C11—H11	119.8	H24A—C24—H24C	109.5
C13—C12—C11	122.05 (18)	H24B—C24—H24C	109.5
C13—C12—H12	119.0	C7—N1—C14	108.48 (13)
C11—C12—H12	119.0	C7—N1—S1	126.97 (12)
C12—C13—C14	117.57 (18)	C14—N1—S1	124.54 (12)
C12—C13—H13	121.2	C22—N2—C21	122.54 (15)
C14—C13—H13	121.2	C22—N2—H2A	118.7
C13—C14—C9	121.21 (17)	C21—N2—H2A	118.7
C13—C14—N1	131.79 (16)	O2—S1—O1	120.59 (11)
C9—C14—N1	107.00 (14)	O2—S1—N1	106.35 (9)
C16—C15—C20	119.43 (19)	O1—S1—N1	106.31 (9)
C16—C15—S2	123.45 (14)	O2—S1—C1	108.84 (11)
C20—C15—S2	117.10 (16)	O1—S1—C1	108.57 (10)
C17—C16—C15	119.7 (2)	N1—S1—C1	105.10 (8)
C17—C16—H16	120.2	C8—S2—C15	103.06 (8)
			
C6—C1—C2—C3	0.3 (3)	C16—C15—C20—C19	0.3 (3)
S1—C1—C2—C3	179.69 (18)	S2—C15—C20—C19	−178.15 (19)
C1—C2—C3—C4	−0.4 (4)	C8—C7—C21—N2	93.9 (2)
C2—C3—C4—C5	0.0 (4)	N1—C7—C21—N2	−85.7 (2)
C3—C4—C5—C6	0.4 (5)	O3—C22—C23—C24	32.6 (4)
C4—C5—C6—C1	−0.4 (5)	N2—C22—C23—C24	−149.7 (2)
C2—C1—C6—C5	0.0 (4)	C8—C7—N1—C14	2.10 (18)
S1—C1—C6—C5	−179.3 (2)	C21—C7—N1—C14	−178.23 (15)
N1—C7—C8—C9	−1.67 (18)	C8—C7—N1—S1	−179.39 (12)
C21—C7—C8—C9	178.66 (16)	C21—C7—N1—S1	0.3 (2)
N1—C7—C8—S2	179.76 (12)	C13—C14—N1—C7	178.38 (18)
C21—C7—C8—S2	0.1 (3)	C9—C14—N1—C7	−1.68 (17)
C7—C8—C9—C10	−178.64 (17)	C13—C14—N1—S1	−0.2 (3)
S2—C8—C9—C10	−0.1 (3)	C9—C14—N1—S1	179.76 (12)
C7—C8—C9—C14	0.63 (18)	O3—C22—N2—C21	−3.8 (3)
S2—C8—C9—C14	179.21 (12)	C23—C22—N2—C21	178.51 (19)
C14—C9—C10—C11	−0.3 (2)	C7—C21—N2—C22	−132.61 (19)
C8—C9—C10—C11	178.90 (17)	C7—N1—S1—O2	25.25 (18)
C9—C10—C11—C12	0.4 (3)	C14—N1—S1—O2	−156.46 (14)
C10—C11—C12—C13	−0.2 (3)	C7—N1—S1—O1	154.92 (15)
C11—C12—C13—C14	−0.1 (3)	C14—N1—S1—O1	−26.79 (16)
C12—C13—C14—C9	0.2 (3)	C7—N1—S1—C1	−90.08 (16)
C12—C13—C14—N1	−179.87 (18)	C14—N1—S1—C1	88.21 (15)
C10—C9—C14—C13	0.0 (2)	C6—C1—S1—O2	−19.4 (2)
C8—C9—C14—C13	−179.39 (15)	C2—C1—S1—O2	161.21 (16)
C10—C9—C14—N1	−179.96 (14)	C6—C1—S1—O1	−152.42 (18)
C8—C9—C14—N1	0.66 (17)	C2—C1—S1—O1	28.22 (19)
C20—C15—C16—C17	0.0 (3)	C6—C1—S1—N1	94.15 (19)
S2—C15—C16—C17	178.35 (17)	C2—C1—S1—N1	−85.20 (17)
C15—C16—C17—C18	0.2 (4)	C7—C8—S2—C15	−93.53 (16)
C16—C17—C18—C19	−0.8 (4)	C9—C8—S2—C15	88.13 (15)
C17—C18—C19—C20	1.1 (5)	C16—C15—S2—C8	20.21 (18)
C18—C19—C20—C15	−0.9 (4)	C20—C15—S2—C8	−161.43 (16)

### Hydrogen-bond geometry (Å, °)

**Table:**

*D*—H···*A*	*D*—H	H···*A*	*D*···*A*	*D*—H···*A*
N2—H2A···O2	0.86	2.52	2.939 (2)	111
C6—H6···O2	0.93	2.57	2.925 (3)	103
C13—H13···O1	0.93	2.37	2.921 (3)	118
C21—H21A···O2	0.97	2.43	2.849 (3)	106
C21—H21B···O3	0.97	2.38	2.767 (2)	103
C18—H18···O2^i^	0.93	2.52	3.317 (3)	144
N2—H2A···O3^ii^	0.86	2.15	2.899 (2)	145
C16—H16···O3^ii^	0.93	2.60	3.416 (3)	147
C10—H10···Cg2^iii^	0.93	2.81	3.658 (2)	152
C5—H5···Cg4^iv^	0.93	2.91	3.788 (4)	158

Symmetry codes: (i) *x*−1, −*y*+1/2, *z*−1/2; (ii) *x*, −*y*+1/2, *z*+1/2; (iii) *x*+1, *y*, *z*; (iv) −*x*, −*y*, −*z*.

## References

[bb1] Bruker (2004). *APEX2* Bruker AXS Inc., Madison, Wisconsin, USA.

[bb2] Chakkaravarthi, G., Dhayalan, V., Mohanakrishnan, A. K. & Manivannan, V. (2008). *Acta Cryst.* E**64**, o392. 10.1107/S1600536807068778 PMC2960260 21201422

[bb3] Chakkaravarthi, G., Ramesh, N., Mohanakrishnan, A. K. & Manivannan, V. (2007). *Acta Cryst.* E**63**, o3564.

[bb4] Nieto, M. J., Alovero, F. L., Manzo, R. H. & Mazzieri, M. R. (2005). *Eur. J. Med. Chem.* **40**, 361–369. 10.1016/j.ejmech.2004.11.008 15804535

[bb5] Olgen, S. & Coban, T. (2003). *Biol. Pharm. Bull.* **26**, 736–738. 10.1248/bpb.26.736 12736524

[bb6] Sheldrick, G. M. (1996). *SADABS*, University of Göttingen, Germany.

[bb7] Sheldrick, G. M. (2008). *Acta Cryst.* A**64**, 112–122. 10.1107/S0108767307043930 18156677

[bb8] Spek, A. L. (2009). *Acta Cryst.* D**65**, 148–155. 10.1107/S090744490804362X PMC2631630 19171970

